# Illuminating the route of precision medicine and inhibitor discovery: real-time measurement of DNA repair capacity with molecular beacons

**DOI:** 10.18632/oncotarget.26056

**Published:** 2018-12-07

**Authors:** Alfonso Bellacosa, Timothy Yen

**Affiliations:** Cancer Epigenetics Program, Fox Chase Cancer Center, Philadelphia, PA, USA; Cancer Biology Program, Fox Chase Cancer Center, Philadelphia, PA, USA

**Keywords:** base excision repair assay, molecular beacon, precision medicine, drug discovery, risk assessment

In this issue, Li et al. applied molecular beacon technology to develop a platform for real-time measurement of DNA repair capacity of cells that has important implications for precision medicine and inhibitor/drug discovery [1].

Base excision repair (BER) is one of the simplest systems of DNA repair in which the damaged (oxidized, alkylated, mismatched, halogenated) base is removed by 1 of 11 human DNA *N*-glycosylases, in a lesion-specific fashion, leaving an apurinic/apyrimidinic (AP) site. Some DNA *N*-glycosylases (defined as bifunctional) can go on and cleave the AP site; otherwise, hydrolytic cleavage of the AP site and consequent strand break is effected by the major AP endonuclease, APE1. Finally, end-trimming enzymes, DNA polymerases, and ligases allow incorporation of correct nucleotide(s), completing the repair reaction [2].

BER alterations play a role in the pathogenesis of cancer and other diseases, including developmental, immune and neurological defects [3]. In addition, BER is involved in active DNA demethylation and transcriptional activation [4]. Importantly, BER capacity affects response to cancer therapy, as evidenced by the clinical relevance of poly (ADP-ribose) polymerase (PARP) inhibitors in the treatment of breast and ovarian cancer [5]. For these reasons, it would be highly beneficial to evaluate BER activity in both normal tissues, to assess disease predisposition, and tumors, to inform cancer therapy.

The conventional methods to measure BER activity are cumbersome because they use gel electrophoresis and often rely on radioactive oligonucleotide substrates. On the other hand, modern next-generation sequencing (NGS) technologies are extremely powerful and useful for the identification of DNA repair defects, when changes in expression levels are dramatic and when the detected nucleotide changes cause frameshift or non-sense mutations. However, these technologies fall short in assessing missense mutations. Even when multiple prediction programs are used and are augmented by careful structural analyses and models, it is difficult to make the call of pathogenic variants vs. non-pathogenic, innocuous polymorphisms [6]. It is clear that for DNA repair proteins, it would be necessary to conduct functional assays that can assess enzymatic activity of the variants.

In the past few years, several methods based on molecular beacons have been developed that promised to provide a rapid assessment of BER activity. These methods employ a hairpin-shaped oligonucleotide substrate, composed of a loop and a damage-containing stem. In the non-cleaved “unrepaired” state, fluorescence of a fluorochrome is quenched by a “black hole” moiety. Upon incubation with purified BER enzymes or cellular extracts, the repair reaction causes strand cleavage which in turn separates the fluorochrome from the “black hole” quencher, leading to a concentration- and time-dependent release of fluorescence that can be monitored in real-time over several hours.

Li et al. provide several improvements to this basic approach. First, they couple the fluorescent dye to a 5’ cytosine, rather than to a 5’ guanine, to avoid the quenching properties of the latter. Second, they conduct the reactions in 0.5 mM EDTA to chelate Mg^2+^ ions and therefore abate fluorescence release by non-specific cellular nucleases. These simple modifications led to a 3-fold increase in signal intensity and background reduction, respectively, with consequent improvement of the signal-to-noise ratio.

The authors also develop a battery of sixteen DNA repair molecular beacons (DRMBs) carrying different types of base damage (and undamaged beacons, as controls) to probe the activity of the 11 human DNA *N*-glycosylases and APE1, in a 96-well plate format. By challenging these DRMBs with nuclear extracts from cancer cell lines, they show that repair activity for any given damage does not necessarily correlate with mRNA levels of the relevant DNA *N*-glycosylases, which further highlights the importance of performing biochemical assays. On the other hand, using purified APE1 preparations, they show that DRMBs are sensitive enough to detect changes in activity stemming from amino acid substitutions that are found in human cancer specimens, which underscores the usefulness of this procedure.

By using DRMBs, Li et al. go on to show conclusively that not only monofunctional, but also bifunctional DNA *N*-glycosylases rely on APE1 for efficient activity, probably for their release from tightly bound product, as proposed in previous studies [7].

This study has several important implications. First, since DNA repair capacity for damage caused by variety of chemotherapeutics is becoming an important component of precision therapy of cancer, by matching anti-cancer drugs to the specific repair defects exhibited by tumor cells [8], the DRMB platform may become an indispensable companion to NGS technologies for molecular characterization of individual tumors and rational development of tailored treatments. To this end, the pilot experiments by Li et al. with bead-anchored DRMBs for fluorescence signal measurement by flow cytometry may accelerate development of this methodology as one of the frontline tools employed by clinical laboratories for precision molecular classification of tumors.

In addition, the robustness and ease of the DRBM methodology may allow its use in the context of precision prevention, i.e. by identifying at-risk individuals that may greatly benefit from primary and secondary prevention interventions because of an inherited defect/reduced activity of a given BER enzyme. In fact, Li et al. show that DRBMs can be utilized to assess repair capacity of peripheral blood mononuclear cells, an easily accessible and routinely isolated source of cellular material, that would be ideal for preventative screens and interventions.

Finally, the DRMBs constitute a powerful technology for inhibitor discovery. A variety of BER enzymes are amplified and/or overexpressed in human cancer, as evidenced by analysis of TCGA data [1]. Thus, for future precision therapy of cancer, the employment of specific inhibitors of BER enzymes could be envisioned as a rational approach to sensitize tumors to certain DNA-damaging therapeutics. This could be a particularly effective strategy to attack cancer stem cells that often overexpress DNA repair proteins and are resistant to DNA-damaging agents. The DRMB approach can be scaled down to the 384- and 1,536-well format, and therefore become amenable to high-throughput screening (Mancuso P, et al., submitted). Using this approach, first-generation inhibitors of the BER enzyme Thymine DNA Glycosylase (TDG) have been isolated, based on the hypothesis that TDG is a novel anti-melanoma target (Mancuso P, et al., submitted). Along these lines, it is easy to imagine that DRMBs can be useful also as a method to evaluate the inhibitory properties of molecules identified by other means.

The utility of DRMBs for assaying BER activity should be expanded to develop assays designed to monitor capacity of other repair systems; newly developed assays could be useful also for screening of inhibitors of other repair pathways.

Time will tell if indeed DRMBs will become an important and routine tool in cancer medicine, but at the moment, they appear to brighten the future for precision oncology and inhibitor discovery.
